# The BRAF kinase domain promotes the development of gliomas *in vivo*

**DOI:** 10.18632/genesandcancer.48

**Published:** 2015-01

**Authors:** Clifford H. Shin, Allie H. Grossmann, Sheri L. Holmen, James P. Robinson

**Affiliations:** ^1^ Huntsman Cancer Institute, University of Utah Health Sciences Center, Salt Lake City, Utah, USA; ^2^ Department of Oncological Sciences, University of Utah Health Sciences Center, Salt Lake City, Utah, USA; ^3^ Department of Pathology, University of Utah Health Sciences Center, Salt Lake City, Utah, USA; ^4^ ARUP Laboratories, Salt Lake City, Utah, USA; ^5^ Department of Surgery, University of Utah Health Sciences Center, Salt Lake City, Utah, USA; ^6^ Hormel Institute, University of Minnesota, Austin, Minnesota, USA

**Keywords:** BRAF, Ink4a/Arf, glioma, mouse model, RCAS/TVA

## Abstract

In-frame BRAF fusions have been observed in over 80% of sporadic pilocytic astrocytomas. In each fusion, the N-terminal autoinhibitory domain of BRAF is lost, which results in constitutive activation via the retained C-terminal kinase domain (BRAF-KD). We set out to determine if the BRAF-KD is sufficient to induce gliomas alone or in combination with *Ink4a/Arf* loss. Syngeneic cell lines demonstrated the transforming ability of the BRAF-KD following *Ink4a/Arf* loss. *In vivo,* somatic cell gene transfer of the BRAF-KD did not cause tumors to develop; however, gliomas were detected in 21% of the mice following *Ink4a/Arf* loss. Interestingly, these mice demonstrated no obvious symptoms. Histologically the tumors were highly cellular and atypical, similar to BRAF^V600E^ tumors reported previously, but with less invasive borders. They also lacked the necrosis and vascular proliferation seen in BRAFV600E-driven tumors. The BRAF-KD-expressing astrocytes showed elevated MAPK signaling, albeit at reduced levels compared to the BRAF^V600E^ mutant. Pharmacologic inhibition of MEK and PI3K inhibited cell growth and induced apoptosis in astrocytes expressing BRAF-KD. Our findings demonstrate that the BRAF-KD can cooperate with *Ink4a/Arf* loss to drive the development of gliomas and suggest that glioma development is determined by the level of MAPK signaling.

## INTRODUCTION

Aberrant RAS mitogen activated protein kinase (MAPK pathway) activation is a hallmark of gliomas [1]. Activation of this pathway occurs through mutations or amplifications in receptor tyrosine kinases or downstream signaling mediators such as RAS, RAF, MEK, and ERK [2]. Recently, a novel mechanism involving *RAF* fusion genes has been identified in pilocytic astrocytomas (PA) that allows for MAPK activation. In-frame fusions between *FAM131B* and *BRAF* have been observed in 2% of sporadic PA [3], fusions between *SRGAP3* and *RAF1* have also been found in 2% of sporadic PA [4], and fusions between *KIAA1549* and *BRAF* have been identified in nearly 80% of sporadic PA samples tested [5–7]. The majority (>70%) of the *KIAA1549:BRAF* fusions occur between exon 16 of *KIAA1549* and exon 9 of *BRAF,* but multiple different fusions have been identified [3,8]. The presence of a BRAF fusion gene is now considered highly diagnostic for PA [9]. These fusions cause anchorage-independent growth when overexpressed in NIH3T3 cells [4,6] and cerebellar neural stem cell (NSC) cultures [10]. Cerebellar engraftment of NSCs expressing *KIAA1549:BRAF* in mice led to the formation of glioma-like lesions after a latency of 6 months [10].

**Figure 1:**
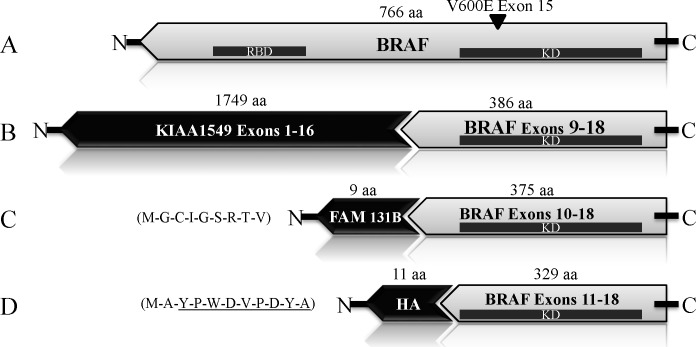
BRAF Schematic A: BRAF^V600E^ B: KIAA1549:BRAF C: FAM131B-BRAF, showing FAM131B amino acids D: BRAF-kinase domain (BRAF-KD), showing amino acids of the HA epitope Tag. RBD=Ras binding domain.

In each fusion the N-terminus of RAF is replaced by FAM131B, SRGAP3 or KIAA1549 resulting in loss of the N-terminal autoinhibitory domain of RAF and constitutive activation of the MAPK pathway via the retained C-terminal kinase domain (BRAF-KD) (Figure 1). The specificity with which the C-terminus of RAF fuses to these different genes suggests that it is required for tumorigenesis in this context; however, the role of the C-terminal domain of *BRAF* within the fusions in glioma formation has not been validated. Expression of a BRAF kinase domain mutant carrying the V600E alteration (BRAF-KD^VE^) was sufficient to induce PA-like lesions in mice [11]. However, in patients, the BRAF kinase domain has not been found to be mutated in this manner in the context of a fusion gene. V600E mutations in full length BRAF are seen in a small percentage of PA (6%) [9,12–14]; however, they are much more common in grade II, and high grade malignant pediatric gliomas; accounting for 18% of grade II, 33% of grade III, and 18% of grade IV tumors (23% grades II-IV) [15]. We have previously demonstrated that *BRAF^V600E^* can cooperate with *Ink4a/Arf* loss to induce high-grade gliomas in mice [16].

The development of small molecule serine-threonine kinase inhibitors (*vemurafenib and dabrafenib)* that specifically target mutant BRAF has revolutionized the treatment of melanoma, and clinical trials are underway for treatment of pediatric gliomas carrying the BRAF^V600E^ mutation (NCT01748149, NCT02034110). However, paradoxically these inhibitors activate MAPK signaling in tumors that do not carry codon 600 mutations, and new small molecule inhibitors designed to break this paradox do not inhibit BRAF fusion mutants at physiologically relevant doses [17]. Furthermore, mutations causing the truncation and loss of the BRAF autoregulatory domain are known to drive resistance to small molecule inhibitors that target the oncogenic codon 600 mutations [10].

In addition to constitutive MAPK activity, mutations targeting the p53/Rb cell cycle pathways are also seen in gliomas. In PA, loss of p16 correlates strongly with reduced senescence, increased cell division, and tumor progression [10,18]. Higher grade pediatric gliomas demonstrate constitutive MAPK activity, but this is almost always accompanied by homozygous deletion of the *cyclin-dependent kinase inhibitor 2A* (*CDKN2A*) locus [13]. This locus encodes both p16 (INK4a) and p14 (ARF) [14,15]. p16 functions to inhibit CDK4/6 mediated phosphorylation of the Retinoblastoma (Rb) protein, and its loss allows for unregulated cell division [19]. Loss of p14 leads to increased ubiquitination and destruction of p53 by HDM2 [20]. Interestingly, 38.5% of PAs show loss of heterozygosity at 9q21, the location of the *CDKN2A* locus and homozygous deletion is seen in 6.4% of cases [21]. A follow-up study of PA patients receiving adjuvant therapy after surgery also found 14% of cases had both p16 loss and BRAF rearrangements [22].

In the current study, we used the well-established RCAS/TVA glioma mouse model to assess the role of the BRAF-KD in glioma development *in vivo*. We show that although the BRAF-KD is not tumorigenic on its own, cooperation with *Ink4a/Arf* loss leads to the development of relatively indolent but highly atypical and cellular gliomas *in vivo*.

## RESULTS

### The BRAF kinase domain promotes anchorage-independent growth

**Figure 2:**
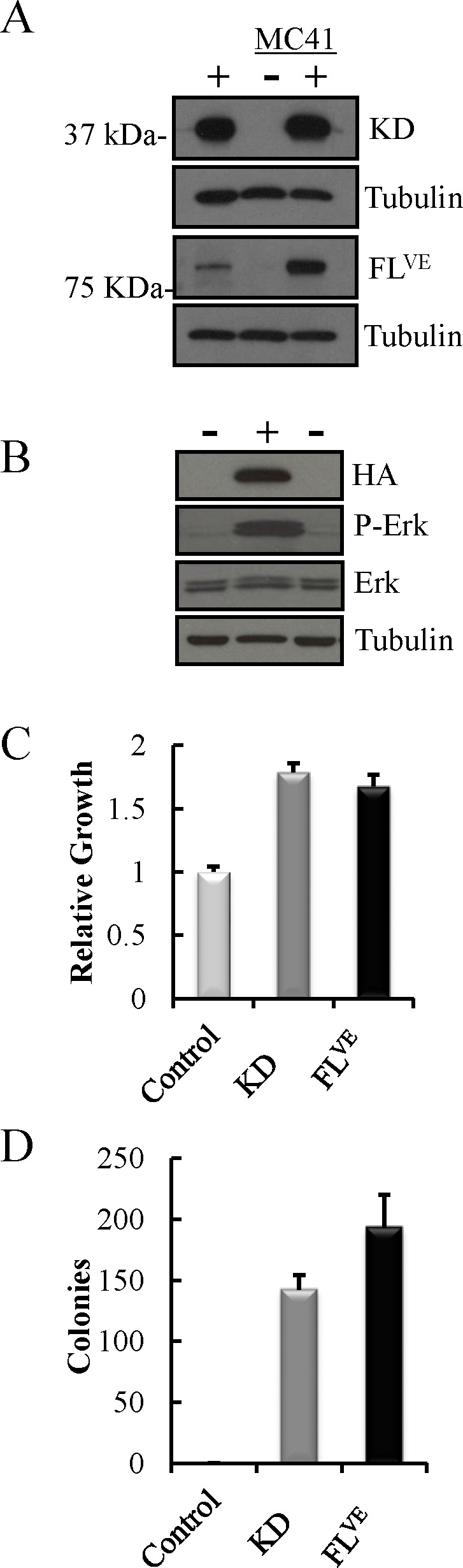
Analysis of the expression and functional activity of the BRAF-KD in the context of Ink4a/Arf-deficiency A: Western blot of *Ink4a/Arf-*deficient astrocytes (MC41+) infected with BRAF-KD (KD) and BRAFV600E (FLVE) with corresponding uninfected, negative control astrocytes (MC41−), and positive control avian fibroblasts (+). B: BRAF-KD expression causes MAPK activation as measured by P-Erk in BRAF-KD transfected human 293FT cells (+) compared to mock transfected cells (−). C: Relative proliferation of *Ink4a/Arf-*deficient astrocytes infected with BRAF-KD (KD) or BRAF^V600E^ (FLVE) compared to uninfected, control cells. The mean of three replicates is shown with error bars for standard error. D: Colony formation of *Ink4a/Arf-*deficient astrocytes infected with BRAF-KD (KD) or BRAF^V600E^ (FL^VE^) compared to uninfected, control cells in soft agar. Error bars reflect standard error of three replicates.

We previously demonstrated the transformative capacity of N-TVA/*Ink4a*/*Arf^−/−^* astrocytes infected with RCASBP(A) viruses containing CRAF*^ß22^*W, BRAF^V600E^ or KRAS*^G12D^*, which form numerous colonies in soft agar [16]. To assess the transforming potential of the BRAF kinase domain BRAF-KD, we infected N-TVA/*Ink4a*/*Arf*^−/−^ astrocytes with RCASBP(A)BRAF-KD and the full length *BRAF* with the V600E mutation, RCASBP(A)BRAFV600E (hereafter BRAF-FL*^VE^*). Gene delivery and expression was confirmed by immunoblotting (Figure 2A). Analysis of phosphorylated Erk 1/2 (P-Erk) demonstrated elevated MAPK activation in cells expressing BRAF-KD (Figure 2B). *Ink4a/Arf*-deficient astrocytes expressing BRAF-KD or BRAF-FL*^VE^* proliferated more rapidly than the negative control; however, there was no significant difference between proliferation of BRAF-KD or BRAF-FLVE cells (P=0.29; Figure 2C). Whereas *Ink4a/Arf-*deficient astrocytes did not form colonies in soft agar, expression of BRAF-KD or our positive control BRAF-FL*^VE^*, induced colonies demonstrating their ability to transform cells in the context of altered Rb and/or p53 signaling (Figure 2D). Interestingly, there was a statistically significant difference between the number of colonies formed by BRAF-KD and BRAF-FL*^VE^* expression (P=0.04).

### The BRAF-kinase domain promotes transformation and tumor growth *in vivo*

A more stringent assay of tumorigenic potential is the ability to form tumors *in vivo*. The cell lines used for these orthotopic studies are syngeneic with the immunocompetent host mouse strain. Therefore, to assess tumorigenicity *in vivo*, 2 × 10^4^ astrocytes were delivered intracranially to newborn mice, and the mice were monitored for signs of tumor formation. *Ink4a/Arf*-deficient astrocytes expressing mutant KRAS served as a positive control. Tumors formed within 3 weeks in 100% of mice injected with *Ink4a/Arf*-deficient astrocytes expressing KRAS^G12D^ (8/8) or the BRAF-KD (8/8). No tumors formed following the injection of uninfected astrocytes within this time frame. Histologically, the BRAF-KD tumors resembled highly cellular and invasive gliomas; both possessed a diffuse growth pattern but lacked the necrosis and endothelial hyperplasia typical of grade IV tumors, glioblastoma multiforme (GBM) (H&E; Supplementary Figure S1). Expression of the BRAF-KD was confirmed using IHC for the HA epitope Tag (HA; Supplementary Figure S1). MAPK pathway activation within the tumors was assessed using IHC for P-Erk (P-Erk; Supplementary Figure S1). All the tumors expressed the glial progenitor marker Nestin; however, expression of the glial fibrillary acidic protein (GFAP) was absent (GFAP, Supplementary Figure S1).

### Expression of the BRAF-KD in combination with *Ink4a/Arf* loss induces gliomas in mice

**Figure 3:**
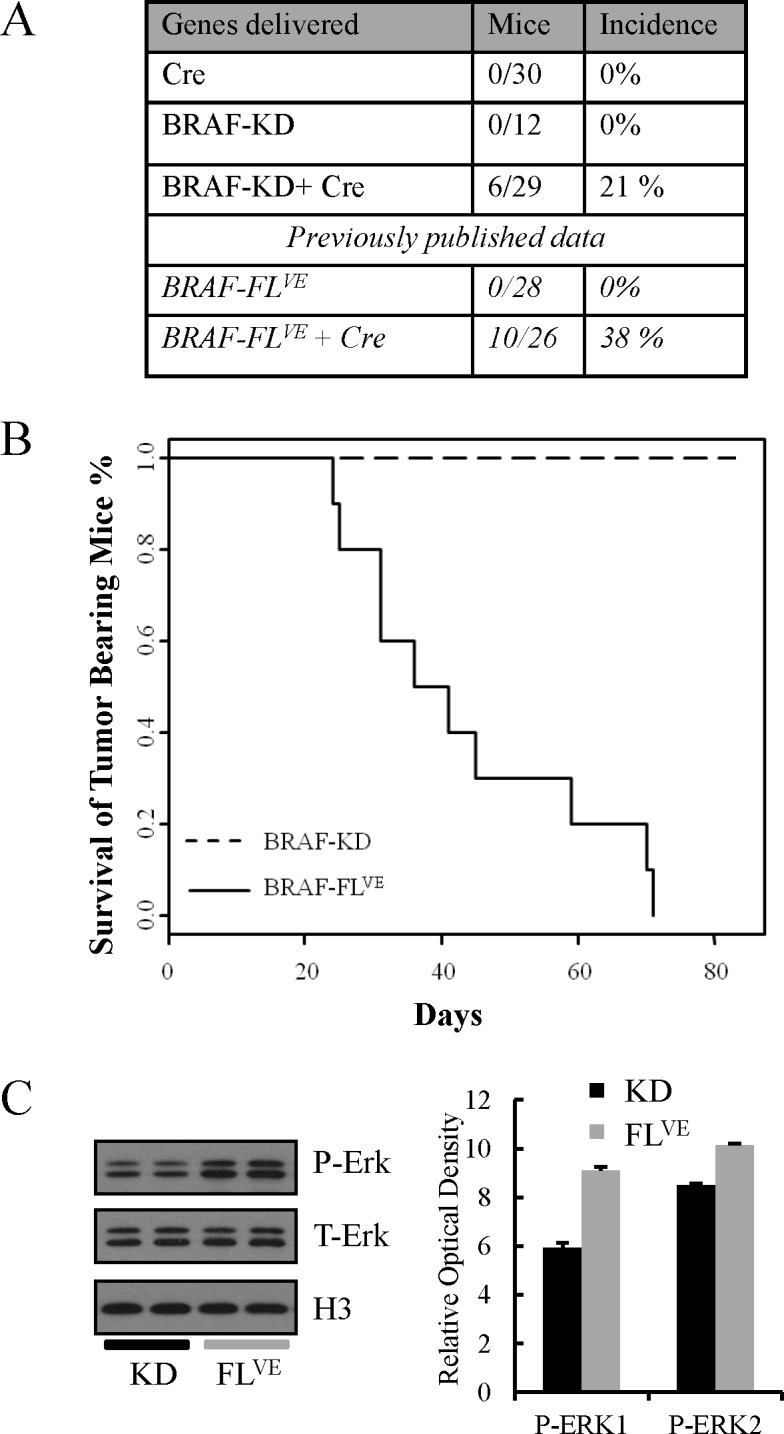
Difference in Survival of Tumor-Bearing Mice between BRAF-KD and BRAF-FLVE A: Incidence of tumor formation for BRAF-KD and BRAF-FL^VE^ in Nestin-TVA;*Ink4a/Arf^lox/lox^* mice. B: Kaplan-Meier post-hoc survival analysis between BRAF-KD *Ink4a/Arf*^−/−^and BRAF-FL^VE^ *Ink4a/Arf*^−/−^ mice. C: Western blot comparing P-Erk levels between BRAF-KD *Ink4a/Arf*^−/−^ and BRAF-FLVE *Ink4a/Arf*^−/−^ astrocytes with corresponding quantification of P-Erk relative to total levels of Erk. Data are represented as mean ± S.E.M of six replicates, of which two are shown in the western blot.

The RCAS/TVA system allows us to deliver genes to somatic Nestin-TVA cells *in vivo.* This enables determination of the roles of specific genes in glioma initiation and progression. Using this system we previously validated the role of mutant BRAF and canonical MAPK signaling in glioma development and maintenance. We found that intracranial infection of newborn N-TVA*/Ink4a/Arf*^lox/lox^ mice with RCASBP(A) viruses containing BRAF-FL^VE^, KRAS^G12D^ or gain of function MEK (MEK^GF^) in combination with Cre leads to the development of high grade gliomas of various subtypes and morphologies [16,27]. Here, we set out to determine if the BRAF-KD is sufficient to induce gliomas *in vivo* alone or in combination with *Ink4a/Arf* loss. Newborn N-TVA*/Ink4a/Arf*^lox/lox^ mice were infected intracranially with RCASBP(A) virus containing BRAF-KD and/or Cre and survival was assessed over a 12 week period. All mice infected with RCASBP(A) viruses containing BRAF-KD (12/12), Cre (30/30) or BRAF-KD + Cre (29/29) survived the 12-week experimental period. Brain tissue from all injected mice was analyzed histologically. None of the mice infected with RCASBP(A) viruses containing BRAF-KD or Cre alone developed tumors; however, gliomas were detected in 21% (6/29) of mice injected with RCASBP(A) viruses containing BRAF-KD and Cre (Figure 3A). This incidence is not statically different (P=0.15) from our previously published experiments where 38% (10/26) of mice injected with full length BRAF^V600E^ and Cre viruses developed tumors; however, the mice with BRAF-FL^VE^-induced tumors had to be sacrificed before 12 weeks due to tumor burden [16] whereas the BRAF-KD mice demonstrated no obvious symptoms (Figure 3B). Histologic analysis of the brains from the BRAF-KD cohort revealed the presence of tumors, which suggests a distinct progression from the BRAF^V600E-^induced tumors. The stark difference in the posthoc survival analysis of tumor-bearing BRAF-KD and BRAF-FL^VE^ mice (P<0.001) as well as the lower level of P-Erk1 and P-Erk2 (P<0.001) in astrocytes expressing BRAF-KD compared to BRAF-FL^VE^ suggests that the BRAF-KD tumors are less malignant gliomas (Figure 3B, C).

**Figure 4:**
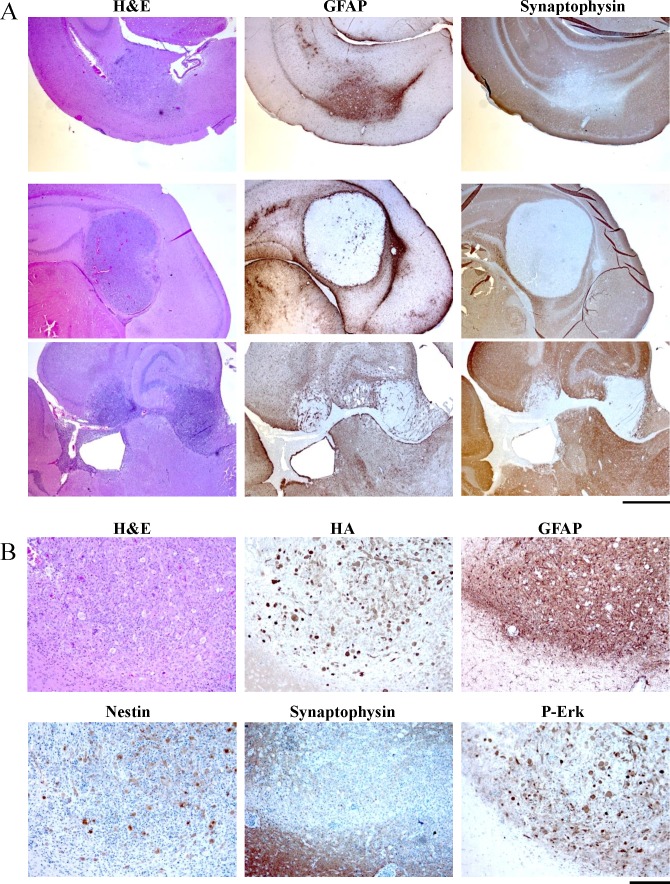
Histological examination of brain sections from RCASBP(A) BRAF-KD and CRE injected mice A: Representative low-power images of brain sections from tumor-bearing mice are shown. H&E: sections stained with hematoxylin and eosin. IHC for synaptophysin demonstrated that none of the tumors were neuronal in origin. Tumor A is representative of tumors that expresses the astrocyte maker GFAP while tumor B is an example that was negative for GFAP expression. Cells in tumor C possessed spindle cell morphology, while tumor B was more epithelioid, and tumor A was mixed as well as containing a population of large non-dividing giant cells. Scale bar represents 1mm. B: High power histological examination of a GFAP expressing tumor. IHC for synaptophysin demonstrates that the tumors are not neuronal and expresses the astrocyte maker GFAP. BRAF-KD expression was detected by IHC for the HA epitope tag on virally delivered BRAF. IHC for P-Erk demonstrates that the MAPK pathway is active in the tumor but not adjacent normal brain. IHC sections were counterstained with hematoxylin. Scale bar represents 200μm.

Histologically, the BRAF-KD *Ink4a/Arf*^−/−^ tumors were highly cellular with atypical nuclei and mitoses, similar to the BRAF-FL^VE^-induced tumors. In contrast to the BRAF-FL^VE^-induced tumors, the BRAF-KD–induced tumors showed a more circumscribed, less invasive border. Furthermore, the BRAF-KD-induced tumors had a lower mitotic index compared to BRAF-FLVE-induced tumors (Figure 4A) [16]. Immunostaining for the cellular proliferation marker Ki-67 demonstrated generally lower cellular proliferation in BRAF-KD tumors compared to BRAF-FL^VE^ tumors which were consistently highly proliferative (Supplementary Figure S2).

Similar to the BRAF-FL^VE^-induced tumors, there was considerable heterogeneity between tumors within the same sections; some were comprised mainly of spindle cells while others were predominantly epithelioid. Several tumors also contained populations of giant tumor cells (Figure 4A). Immunostaining using a C-terminal antibody for BRAF and for the HA-epitope tag confirmed expression of virally delivered BRAF-KD. All of the tumors expressed Nestin, and GFAP expression was present in several smaller tumors *(Figure 4B)*. Together, the lower mitotic index, the lower MAPK activation, and the more circumscribed tumor borders may explain the lack of symptoms observed in these mice at 12 weeks post-injection as compared to BRAF-FLVE-induced tumors. These distinct features suggest that the tumors driven by BRAF-KD have a later onset or more indolent behavior than tumors driven by BRAF^V600E^ [16].

### MEK and PI3K/mTOR inhibition reduces proliferation and induces significant apoptosis in cells expressing the BRAF-KD

**Figure 5:**
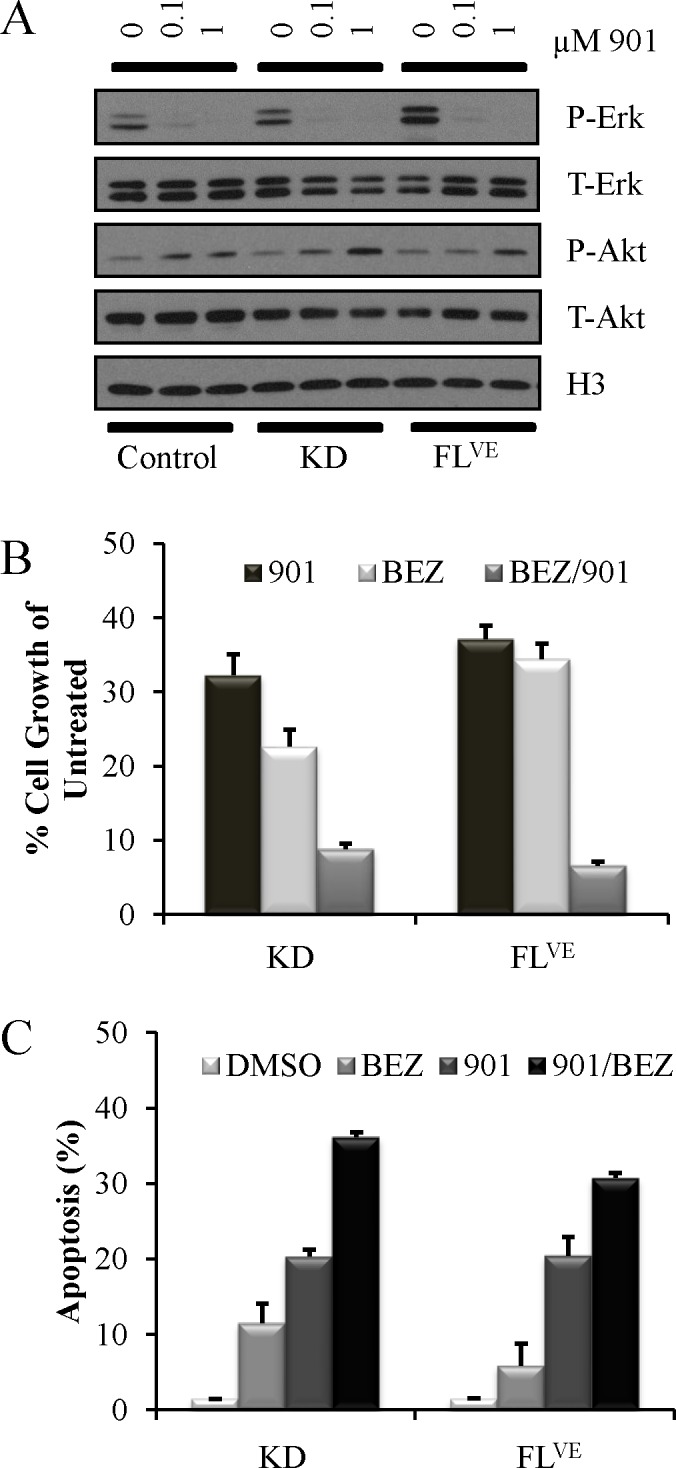
Pharmacological inhibition of cells harboring mutant BRAF A: MEK Inhibition with PD0325901 at 0 μM, 0.1 μM, and 1.0 μM for one hour in RPMI 2% FBS of *Ink4a/Arf-*deficient astrocytes infected with BRAF-KD (KD) and BRAF^V600E^ (FL^VE^) with corresponding uninfected, negative control astrocytes. MEK inhibition decreases P-Erk levels by western blot. B: Growth inhibition in *Ink4a/Arf-*deficient astrocytes infected with BRAF-KD (KD) and BRAF^V600E^ (FL^VE^) treated in triplicate with 1 μmol/l PD0325901 (901), 1 μmol/l NVP-BEZ235 (BEZ) or combination (BEZ/901) for 96 h. Cells treated with vehicle alone (DMSO) served as controls. Cell growth was measured using the ATPlite assay. Data was normalized to untreated controls and are represented as mean ± S.E.M. C: Apoptosis induced by inhibition of MEK and PI3K/mTOR signaling in mouse astrocytes. *Ink4a/Arf-*deficient astrocytes infected with BRAF-KD (KD) and BRAF^V600E^ (FL^VE^) were treated in triplicate with either DMSO as a control, 1 μmol/l PD0325901 (901), 1 μmol/l NVP-BEZ235 (BEZ) or in combination (BEZ/901) for 96 h. Apoptosis was quantitated using Guava ViaCount. Data are represented as mean ± S.E.M.

Type 1 BRAF^V600E^ mutant inhibitors (vemurafenib, dabrafenib) have been demonstrated to cause paradoxical activation of the MAPK pathway in cells expressing the BRAF-KD fusions [17]. Since activating mutations in *BRAF* drive cell growth and proliferation primarily through canonical MAPK signaling (e.g., BRAF-MEK*–* ERK), we chose to test the effect of Mek inhibition on the growth of cell lines driven by BRAF-FL^VE^ and BRAF-KD. The concentration required for the inhibition of Erk phosphorylation with the Mek inhibitor PD0325901 (hereafter 901) in N-TVA;*Ink4a/Arf*^−/−^ astrocytes expressing BRAF-FL^VE^ and BRAF-KD, was determined by titration using 100 nmol/L and 1 μmol/L of 901. Treatment with 0.1μM significantly reduced Erk1/2 phosphorylation and 1 μM of the Mek inhibitor was sufficient for complete blockade (Figure 5A). As expected, we observed a negative-feedback loop between Mek and PI3K signaling – evidenced by the increasing levels of P-Akt with increasing Mek inhibition [27]; therefore, we also targeted PI3K/mTOR signaling using 1 μmol/l of the PI3K/mTOR inhibitor NVP-BEZ235 (hereafter; BEZ). The proliferation of *Ink4a/Arf*^−/−^ astrocytes expressing BRAF-FL^VE^ and BRAF-KD was significantly inhibited by both 901 and BEZ. Combined treatment with 901 and BEZ produced an additive effect (S=1.2, P=0.0633) in the context of the BRAF-KD and synergistic effect in the context of BRAF^V600E^ (S= 0.516, P=0.0001) as assessed by reduction in cell growth over 96 hours with synergy and additivity calculations performed as described previously (Figure 5B) [27]. A significant increase in apoptosis in BRAF^V600E^ and BRAF-KD cells was also observed (Figure 5C).

## DISCUSSION

While the role of the N-terminal segment within BRAF fusion genes (i.e. KIAA1549, SRGAP3, FAM131b) is unknown, there is evidence to suggest their possible function. SRGAP3 has been proposed to play a role in neurogenesis [28] and contains a Fes/CIP4 Homology (FCH) domain, which has a putative function in regulating the microtubule cytoskeleton [29]. KIAA1549 harbors two transmembrane domains, which have been proposed to tether RAF to the membrane. However, the minimal FAM131b fusion component is only 9 amino acids in size and has no known or predicted function [3]. Our results demonstrate that the BRAF-KD alone can cooperate with *Ink4a/Arf* loss to drive gliomagenesis *in vivo*. Deletion of the N-terminal auto-inhibitory domain allows activation of the protein at sufficient levels to drive tumorigenesis. We suspect that the transforming capabilities of the various BRAF fusion genes lies within the BRAF-KD, and the promoters of FAM131b, SRGAP3, and KIAA1549 simply serve to direct expression to the appropriate cells in the brain. Thus, the result of the rearrangements is the loss of the regulatory domain leading to constitutive MAPK activation, a phenomenon that is observed in 80-90% of PAs [30]. While it has been shown before that BRAF-KD^VE^ can form lesions in mice resembling PA [11], BRAF fusion genes in humans do not harbor the V600E mutation. To our knowledge, this is the first report to show that BRAF-KD can lead to glioma development in cooperation with *Ink4a/Arf* loss.

Expression of a powerful oncogene, such as BRAF^V600E^ in otherwise normal cells can prevent proliferation by inducing senescence; this phenomenon, referred to as ‘Oncogene Induced Senescence’ (OIS), is especially common in melanoma where it prevents the progression of benign nevi with BRAF^V600E^ mutations into melanoma [31]. Loss of p16 expression is a common occurrence in melanoma and interestingly loss of p16 also correlates strongly with reduced senescence, increased cell division, and progression of PAs into WHO grade II astrocytomas [22,32]. The BRAF-KD *Ink4a/Arf*^−/−^ mice demonstrated no obvious symptoms within the 12-week experimental timeframe and survived to endpoint in contrast to the BRAF-FL^VE^ *Ink4a/Arf*^−/−^ mice that began to die from the 3rd week. Other distinguishing characteristics between the BRAF-KD and BRAF-FL^VE^ include histology showing a lack of necrosis and less invasive borders as well as immunoblotting demonstrating decreased levels of MAPK activation in tumors driven by the BRAF-KD. This suggests that the level of canonical BRAF signaling plays a role in glioma development and progression. Fascinatingly, expression of the full length KIAA1549:BRAF fusion protein has never been detected either *in vitro* or *in vivo,* even in KIAA1549:BRAF transgenic mice [33], suggesting that sporadic PA require low levels of BRAF signaling, and that this is one way in which they may escape OIS in the context of p16 expression. Hereditary melanoma–astrocytoma syndrome is characterized by a dual predisposition to melanoma and development of low-grade paediatric astrocytomas. This syndrome is associated with germline mutations that effect only *CDKN2A* exon 1β, resulting in loss of p14 expression but not p16 expression [34]. For these reasons, future experiments will concentrate on determining the grade and response to treatment of tumors induced with BRAF-KD and either p16 or p14 loss.

MEK inhibition in gliomas is an area of ongoing study. Currently, a clinical trial (NCT02124772) is underway to investigate the efficacy of treating V600E gliomas with a combination of a Trametinib and Dabrafenib. Recently, both drugs have shown limited penetration into the brain due to active efflux across the blood-brain-barrier (BBB) [35,36]. Thus for our *in vitro* studies, we opted for 901 because it has been shown to penetrate the BBB [37,38]. In fact the toxic effects of 901 are likely due to its ability to cross the BBB. The paradoxical activation of the MAPK pathway caused by BRAF^V600E^ inhibitors could be inhibited by dual BRAF-MEK inhibition as has been observed in melanoma, but the consequence of this is expected to be more p-AKT [39]. As proposed previously, we believe that a combination of 901 and BEZ can serve as a rational cancer therapeutic [27]. To date there have been no clinical trials studying the specific combination of BEZ and 901; however, two trials have examined the combined effect of dual inhibition MAPK and PI3K involving either BEZ or 901. NCT01337765 was a phase 1 trial for studying BEZ with a different MEK compound (MEK162), and NCT01347866 is a currently ongoing phase 1 trial to study 901 with another PI3K-mTOR compound (PF-05212384).

In summary, our findings demonstrate that the BRAF-KD region of the numerous BRAF fusion genes drives gliomagenesis. As cancers with BRAF mutations are often addicted to this signaling pathway, targeting BRAF signaling represents a reasonable therapeutic strategy. However, cells expressing KIAA1549-BRAF fusions genes display paradoxical activation when they are targeted with BRAF^V600E^ inhibitors (i.e. vemurafenib) [40]. Unfortunately, next generation, paradox breaker BRAF inhibitors that have reduced capacity to paradoxically activate wild-type BRAF do not inhibit BRAF fusions genes at physiologically relevant doses [17,41]. We previously demonstrated that efficacy of combined MEK and PI3K/mTOR inhibition for the treatment of high-grade gliomas driven by BRAF^V600E^ [27]; our latest results suggest that combined MEK and PI3K/mTOR inhibition is a rational therapy for the treatment of gliomas driven by fusion genes containing the BRAF-KD as well.

## METHODS

### Mice and genotyping

Nestin-TVA;*Ink4a/Arf^lox/lox^* mice and genotyping procedures have been described [16]. The mice are on a mixed genetic background consisting of FVB/n, 129, and C57Bl/6. PCR genotyping for the TVA transgene and for the *Ink4a/Arf^lox^* and wild-type *Ink4a/Arf* alleles was performed as described [16,23]. All experiments were approved by the IACUC before experimentation.

### Establishment of *N-TVA;Ink4a/Arf*^lox/lox^ astrocytes in culture

N-TVA;*Ink4a*/*Arf*^lox/lox^ primary astrocytes were established following dissection of newborn mouse brain tissue by physical disruption into single cells using scalpels and 0.25% trypsin. Cell cultures were maintained in RPMI (Invitrogen) containing 10% FBS, and gentamicin at 37°C.

### Viral constructs

The retroviral vectors used in this study are replication-competent avian leukosis virus (ALV) long terminal repeat (LTR), splice acceptor, and Bryan polymerase-containing vectors of envelope subgroup A [designated RCASBP(A) and abbreviated RCAS]. RCASBP(A)CRE, RCASBP(A)BRAF^V600E^ and RCASBP(A)KRAS^G12D^ have been previously described [16,23]. RCASBP(A)BRAF-KD was created by PCR amplification from pcDNA3.1 BRAF template DNA, [a gift from Martin McMahon (UCSF)] followed by TOPO cloning into the Gateway entry vector pCR8 (Invitrogen) and recombination into RCASBP(A) DV using LR Clonase Enzyme Mix (Invitrogen) (Forward: atggcgtacccatacgacgtcccagactacgctaaaacactt ggtagacgggactc, Reverse: cagtggacaggaaacgcacca) per the manufacturer's specifications. To propagate the RCAS viruses, proviral DNA was transfected into DF-1 cells grown at 39°C using the calcium phosphate transfection method [24]. RCAS vectors are replication-competent in the DF-1 cell line, an immortalized chicken fibroblast line, and high titer viral stocks can be obtained [25]. The supernatants were filtered through a 0.45-μm filter, and viral titers were determined as described [26].

### Viral infections *in vitro*

Primary N-TVA;*Ink4a*/*Arf*^lox/lox^ astrocytes were seeded in 6-well plates at a density of 5 × 10^4^ cells/well and were maintained in RPMI with 5% FBS, gentamicin at 37°C as described previously [16]. After the cells attached, 1 mL of filtered virus-containing medium was added in the presence of 8 μg/ml polybrene (Sigma) for 2 h at 37°C in 5% CO_2_.

### Growth in soft agar

To assess anchorage-independent growth, 1.5 × 10^5^ cells were suspended in 0.35% Difco agar noble (Becton Dickinson) in RPMI with 10% FBS and layered over pre-solidified 0.65% Difco Noble Agar in RPMI with 10% FBS per well of a six-well dish. Each cell line was assayed in triplicate.

### Western blotting

Protein concentrations were determined using the Bio-Rad D Protein Assay (Bio-Rad). The proteins were separated on a 4-20% Tris-glycine gradient polyacrylamide gel, transferred to nitrocellulose, and incubated for 1 h at room temperature in blocking solution (0.05% Tween-20 in Tris-buffered saline with 5% NFDM or 5% BSA). Blots were immunostained for the following antigens: phospho-Erk at Thr202/Tyr204 (4370, 1:1000, Cell Signaling); total Erk (9102, 1:1000, Cell Signaling); tubulin (T9026, 1:5000, Sigma); HA epitope on BRAF-KD (HA.11, 1:1000, Covance); C-terminus of BRAF (SC166, 1:500*,* Santa Cruz Biotechnology); and BRAF^V600E^ (E1929, 1:2000, Spring Bioscience). The blots were then incubated with an anti-mouse or anti-rabbit IgG-HRP secondary antibody, incubated with ECL solution (Amersham), and exposed to film (Kodak).

### *In vivo* infection

Infected DF-1 cells from a confluent culture in a 10-cm dish were trypsinized, pelleted, resuspended in 50 μL PBS, and placed on ice. Newborn mice were injected intracranially 2 mm ventral from bregma (intersection of the coronal and sagittal sutures) with 5 μL of infected DF-1 cells using a gas-tight Hamilton syringe as described previously [16,27].

### Histological analysis

Brain tissue from injected mice was fixed in 10% formalin, embedded in paraffin and 5-μm sections were adhered to glass slides. The sections were stained with hematoxylin and eosin or left unstained for immunohistochemistry (IHC). Images were captured using a Zeiss Axio microscope equipped with an AxioCam ICc3 camera (Zeiss).

### Immunohistochemistry

Tissue sections were de-paraffinized and antigen retrieval was performed in ‘Diva Decloaking’ buffer (Biocare Medical) by boiling for 10 min. Sections were treated with 3% H_2_O_2_ and blocked in Background Sniper (Biocare Medical) for 10 min. Primary antibodies were diluted in Renaissance background reducing diluent (Biocare Medical). Sections were incubated overnight at 4°C and probed with Mach 4 rabbit polymer reagent (Biocare Medical) or Mach 4 mouse probe for 15 min followed by Mach 4 polymer for 15 min for mouse monoclonal antibodies. Visualization was carried out with DAB (Biocare Medical). Sections were counterstained with hematoxylin. Antibodies against the following antigens were used: GFAP (13-0300, 1:500, Invitrogen); synaptophysin (ab32127, 1:200, Abcam); BRAF-KD (detected using an antibody to the HA epitope (HA.11, 1:1000, Covance) and C-terminal BRAF antibody (SC166, 1:500, Santa Cruz Biotechnology); Nestin (ab6142, 1:200, Abcam); phospho-Erk (4370, 1:100, Cell Signaling); and Ki-67 (M7249, 1:50, Dako) using a rabbit anti rat linker (P0450, 1:50, Dako)

### Drug Treatment

To measure the effect of MEK inhibition on phosphorylated Erk (P-Erk) in astrocytes expressing BRAF constructs, drug was serially diluted in 10 fold increments in RPMI containing 2% FBS and added to cells in 6-well plates. 0.1% DMSO vehicle was added in negative control wells. After 1 h, cells were washed twice in PBS before analysis by Western blot. To measure the effect of the MEK inhibitor PD0325901 or the PI3K/mTOR inhibitor NVP-BEZ235 on cell proliferation as measured by ATP levels, drug was diluted to 1.0 μM and added to astrocytes. After 96 h, ATP levels were detected using the ATPlite 1step Luminescence Assay System (Perkin Elmer) following the manufacturer's instructions. In brief, 100μl of reconstituted ATPlite reagent was added to cells in a 96-well plate. The plate was incubated on an orbital shaker at 700rpm for 3 min and dark-adapted for 10 min before luminescence was read for 0.1 sec per well on a Synergy HT multi-mode microplate reader (BioTek). To measure the effect of the MEK inhibitor PD0325901 or the PI3K/mTOR inhibitor NVP-BEZ235 on apoptosis, drugs were diluted to 1.0 μM and added to astrocytes. After 96 h, the cells were resuspended in Guava Viacount reagent (Millipore) and 5000 events per sample were read according to the manufacturer's instructions as described previously [27]. All drug treatment experiments were done in three biological replicates.

### Statistical Analysis

Western blot density analysis was performed using Image J. Kaplan Meier survival data analysis and determination of synergy between PD032509 and NVP-BEZ235 were done as previously described [27]. To compare means, two-tailed Student's t test was used. P values below 0.05 were considered significant.

## SUPPLEMENTARY MATERIAL FIGURES


