# Supplementary material for: The BRAF kinase domain promotes the development of gliomas in vivo
Source: Genes Cancer. 2015 Jan;6(1-2):9–18. doi: 10.18632/genesandcancer.48 (PMC4362480; doi:10.18632/genesandcancer.48)
Supplement: Supplementary file 1 [file ganc-06-009-s001.pdf]

The BRAF kinase domain promotes the development of gliomas *in vivo*

Supplementary Material

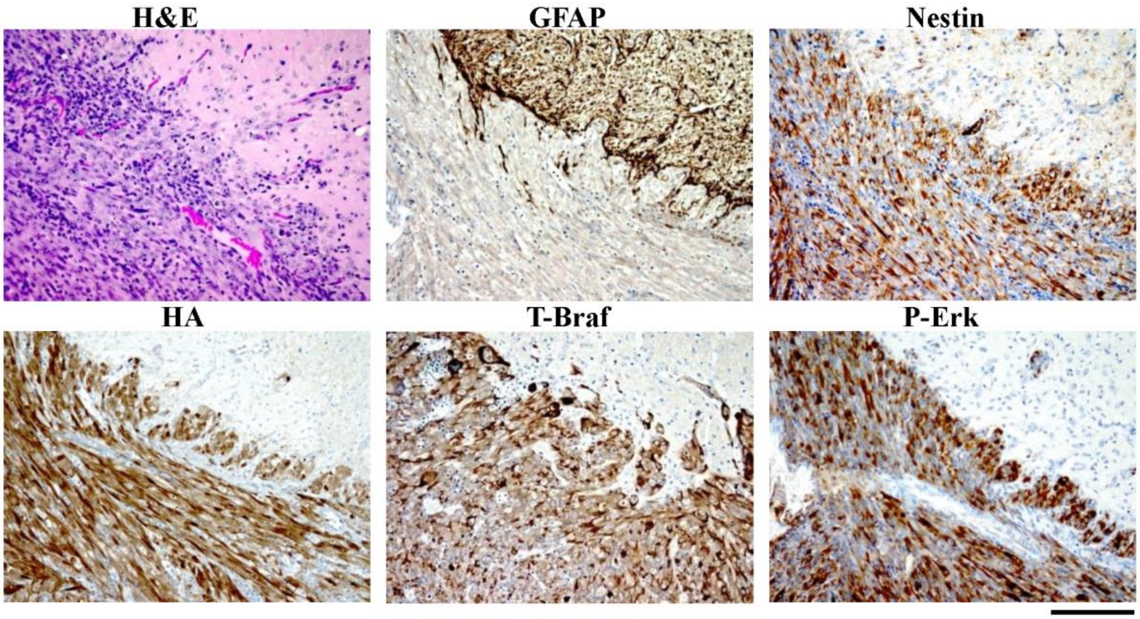

Supplementary Figure S1: Histological examination of brain sections from mice injected with *Ink4a/Arf*-deficient astrocytes expressing BRAF-KD. H&E: sections stained with hematoxylin and eosin. IHC for P-Erk demonstrates that the MAPK pathway is active in the tumor but not adjacent normal brain. Nestin and GFAP are glial specific markers. BRAF-KD expression was detected by IHC for the HA epitope tag on virally delivered BRAF or by using the C-terminal BRAF antibody. IHC sections were counterstained with hematoxylin. Scale bar represents 200μm.

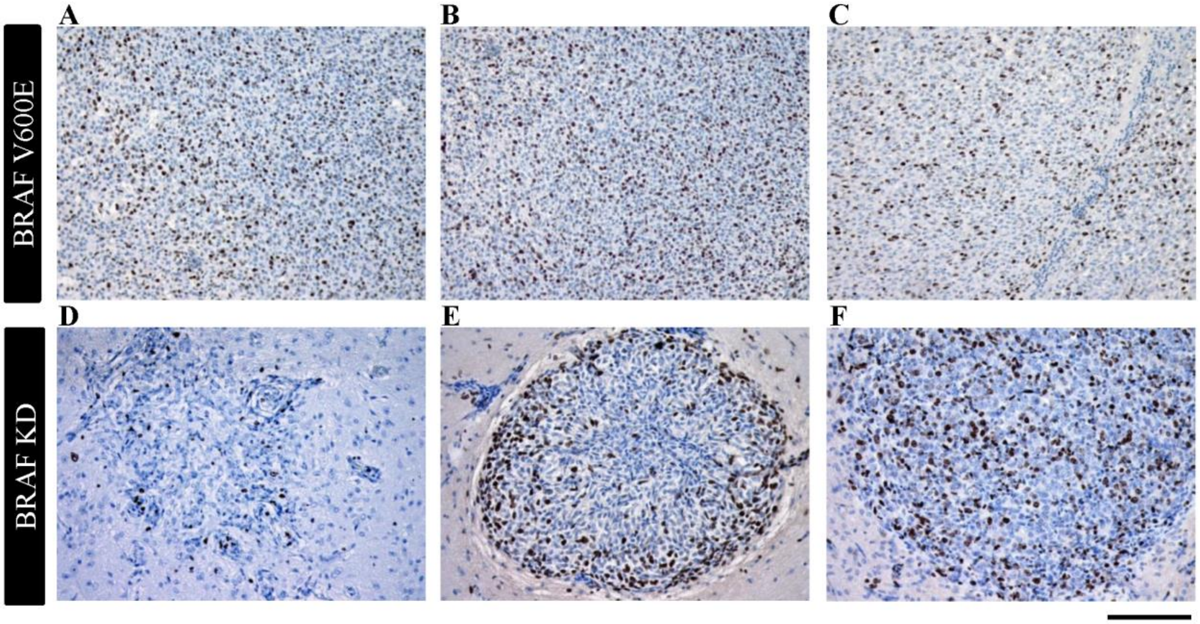

Supplementary Figure S2: A comparison of cellular proliferation between brain sections from RCASBP(A) BRAF-KD & CRE injected and RCASBP(A) BRAFV600E & CRE injected mice. IHC for cellular proliferation marker Ki67 demonstrated that BRAFV600E tumors (A-C) were consistently highly proliferative while proliferation varied significantly in BRAF-KD tumors (D-F). IHC sections were counterstained with hematoxylin. Scale bar represents 200μm.
